# Variation in reward quality and pollinator attraction: the consumer does not always get it right

**DOI:** 10.1093/aobpla/plv034

**Published:** 2015-04-09

**Authors:** David E. Carr, Ariela I. Haber, Kathryn A. LeCroy, De'Ashia E. Lee, Rosabeth I. Link

**Affiliations:** 1 Blandy Experimental Farm, University of Virginia, 400 Blandy Farm Lane, Boyce, VA 22620, USA; 2 Department of Environmental Sciences, University of Virginia, Charlottesville, VA 22904, USA; 3 Department of Infectious Diseases, University of Georgia, 500 DW Brooks Drive, Athens, GA 30602, USA; 4 Department of Entomology, University of Wisconsin, Madison, WI 53706, USA

**Keywords:** *Bombus impatiens*, floral reward, honest signal, inbreeding, *Mimulus guttatus*, olfaction, pollen quality, pollinator preference

## Abstract

Nearly all bees rely on pollen as the sole protein source for the development of their larvae. The central importance of pollen for the bee life cycle should exert strong selection on their ability to locate the most rewarding sources of pollen. Despite this importance, very few studies have examined the influence of intraspecific variation in pollen rewards on the foraging decisions of bees. Previous studies have demonstrated that inbreeding reduces viability and hence protein content in *Mimulus guttatus* (seep monkeyflower) pollen and that bees strongly discriminate against inbred in favour of outbred plants. We examined whether variation in pollen viability could explain this preference using a series of choice tests with living plants, artificial plants and olfactometer tests using the bumble bee *Bombus impatiens*. We found that *B. impatiens* preferred to visit artificial plants provisioned with fertile anthers over those provisioned with sterile anthers. They also preferred fertile anthers when provided only olfactory cues. These bumble bees were unable to discriminate among live plants from subpopulations differing dramatically in pollen viability, however. They preferred outbred plants even when those plants were from subpopulations with pollen viability as low as the inbred populations. Their preference for outbred plants was evident even when only olfactory cues were available. Our data showed that bumble bees are able to differentiate between anthers that provide higher rewards when cues are isolated from the rest of the flower. When confronted with cues from the entire flower, their choices are independent of the quality of the pollen reward, suggesting that they are responding more strongly to cues unassociated with rewards than to those correlated with rewards. If so, this suggests that a sensory bias or some level of deception may be involved with advertisement to pollinators in *M. guttatus*.

## Abstract (precis)

Bees depend on pollen as the primary protein source for their larvae and should be strongly selected to identify cues associated with the most rewarding flowers. We examined the ability of bumble bees (*Bombus impatiens*) to identify the most rewarding foraging opportunities using arrays of live monkeyflowers (*Mimulus guttatus*), artificial plants, and pairwise olfactory tests. Bees could identify pollen rewards by scent and tended to visit the most rewarding artificial flowers. They seemed less able to identify the best pollen sources when foraging on live plants. We suggest that live plants may provide conflicting or deceptive signals to pollinators.

## Introduction

Plant–pollinator interactions are typically viewed as mutualisms, with pollinators transferring pollen to stigmas and facilitating plant reproduction while they forage for floral resources such as nectar and pollen. Pollen is a particularly important resource for bees because it serves as their primary protein source and is required by nearly all bees to rear their larvae and to develop the ovaries of egg-laying females (Michener 2000). Variation in protein content of larval provisions has been demonstrated to affect larval and adult bee body size (Roulston and Cane 2002; Vanderplanck *et al*. 2014) and longevity (Li *et al*. 2014). As consumers in a pollen-based economy, bees should be under strong selection to recognize cues associated with pollen rewards that could allow them to maximize their efficiency by being discriminating foragers. Despite the central importance of pollen in the ecology of bees, there has been surprisingly little attention paid to the importance of variation in pollen rewards in explaining their foraging decisions.

The amount of protein in pollen varies widely across species, and there is some evidence that bee-pollination has influenced the evolution of protein content (Roulston *et al*. 2000; Hanley *et al*. 2008). Evidence that bees choose plant hosts on the basis of the protein content of their pollen is more equivocal (Roulston *et al*. 2000; Hanley *et al*. 2008; Vanderplanck *et al*. 2014). A few studies have examined intraspecific variation in pollen rewards. Many studies have demonstrated that pollinating bees prefer male fertile plants over females in gynodioecious and dioecious species (reviewed by Vega-Frutis *et al*. 2013). Olfactory cues provide one means of discrimination, and Ashman *et al*. (2005) demonstrated that pollinator preference for male fertile over female wild strawberries (*Fragaria virginiana*) was primarily due to volatiles emitted from the anthers. Robertson *et al*. (1999) demonstrated that bumble bees (*Bombus* spp.) selectively foraged on *Mimulus guttatus* with high male fertility in both pairwise choice tests between greenhouse plants and in field trials with patches of plants that varied in pollen viability. Protein content is positively correlated with pollen viability in *M. guttatus* (Yeamans *et al*. 2014), suggesting that this preference may be adaptive.

Bumble bees preferentially visit outbred *M. guttatus*, discriminating against inbred plants (Ivey and Carr 2005; Carr *et al*. 2014). Inbreeding reduces pollen production and pollen viability in *M. guttatus* (Carr and Dudash 1995, 1997; Willis 1999), lowering the quality of their rewards. Yeamans *et al*. (2014) found an 11-fold difference in total protein mass per flower between the inbred plant with the lowest pollen viability and the outbred plant with the highest pollen viability. Although the observed preference for outbred plants would certainly be consistent with expectations for optimizing the collection of protein rewards, Carr *et al*. (2014) found that pollen viability explained significant variation in visitation rates in only one of the two *M. guttatus* populations under study. Although the importance of variation in pollen viability in attracting pollinator visits in that population was essentially equivalent to the importance of variation in floral display size, it explained only 8 % of the variation in visitation rates.

Here we report on further studies with *M. guttatus* to test whether bumble bees are capable of making foraging decisions based on variations in pollen rewards. The genus *Mimulus* (Phrymaceae) has proved extremely useful for studies of inbreeding and mating systems for over 130 years (e.g. Darwin 1878) and more recently has proved useful in studying novel aspects of plant–insect interactions (Schemske and Bradshaw 1999; Levine 2000; Carr and Eubanks 2002; Hare 2002; Bradshaw and Schemske 2003; Ivey *et al*. 2004; Elderd 2006; Karron *et al*. 2006; Bodbyl Roels and Kelly 2011; Holmquist *et al*. 2012; Meindl *et al*. 2013; Rae and Vamosi 2013; Byers *et al*. 2014*a*, *b*; Carr *et al*. 2014; Grossenbacher and Stanton 2014). This diverse genus is native throughout the Americas and includes transitions between pollination syndromes, mating systems and life-history patterns with a well-resolved phylogeny (Beardsley *et al*. 2004). A secondary centre of diversity occurs in Australia (Beardsley and Barker 2005). Many of the species have relatively short life spans and are easily cultured and crossed, making them highly suitable for manipulative experiments. Extensive genetic tools have been developed for *M. guttatus* and its close relatives, *M. lewisii* and *M. cardinalis* (Wu *et al*. 2008).

In this series of studies, we attempted to disentangle the bumble bee's preference for outbred *M. guttatus* and possible preferences for flowers offering the greatest pollen rewards. To do this, we used a combination of experiments with live plants, artificial plants provisioned with fresh anthers and olfactometer tests, taking advantage of outbred subpopulations developed in our lab that show high levels of male sterility. Specifically we ask: (i) Will bumble bees discriminate against outbred plants that have low pollen viability? (ii) In the absence of other floral cues, are bumble bees able to discriminate between anthers with low or high viability? (iii) In the absence of other floral cues, are bumble bees able to discriminate between anthers from inbred and outbred plants? (iv) Can bumble bees discriminate between fertile and sterile anthers based only on olfactory cues? (v) Can bumble bees discriminate between inbred and outbred plants based only on olfactory cues?

## Methods

### Study system: *Mimulus guttatus* and *Bombus impatiens*

*Mimulus guttatus* (seep monkeyflower) is native to western North America from northern Mexico to Alaska. Most populations are annual, but in some coastal areas or in areas that are continuously wet, it can be perennial. It is self-compatible, and populations range widely in outcrossing rates. Its large, yellow, zygomorphic flowers are visited by many different insects, but bumble bees (*Bombus* spp.) are thought to be the most important pollinators. The flowers produce little or no nectar, so pollen appears to be the primary, if not exclusive, reward to pollinators.

The *M. guttatus* used in this study were derived from a large population in Napa County, CA, USA (38°42′06″N, 122°24′29″W). Seeds were collected from over 500 random maternal families in spring 2007 while walking along ∼500 m of stream bank. One plant from each maternal family was randomly crossed in a greenhouse at Blandy Experimental Farm (Boyce, VA, USA) to produce a fully outbred base population. The plants used in the studies described here were derived from an unrelated study on the response to selection. In that study, two random samples (groups A and B) of 150 of these randomly crossed families were used to start experimental subpopulations. Five subpopulations of 50 individuals were derived from each of these samples: IL—an inbreeding (selfing) population selected for reduced stigma-anther separation and low leaf trichome density, IH—an inbreeding population that had been selected for reduced stigma-anther separation and high trichome density, OL—a fully outbred population (with no biparental inbreeding) that had been selected for increased stigma-anther separation and low trichome density, OH—a fully outbred population that had been selected for increased stigma-anther separation and high trichome density and OC—a control population that was randomly outbred but under no selection. Each subpopulation passed through four generations of selection (or no selection in the case of the control), so that the inbred plants had an inbreeding coefficient of *f* = 0.9375. Outcrossed plants were produced by randomly pairing parents with the constraint that parents could not share any ancestors in their pedigree dating back to the original random crosses derived from field-collected seed. This resulted in plants with no biparental inbreeding. Outbred and inbred crosses were made by collecting an anther from the pollen donor with a jeweler's forceps and rubbing the anther directly onto the stigma of the pollen recipient.

Inbreeding subpopulations (IL and IH) had significantly smaller corolla widths and lengths relative to outbred populations (OC, OH and OL) (D. E. Carr and M. D. Eubanks, unpubl. data). The trichome selection regimes, however, did not alter corolla length or width (D. E. Carr and M. D. Eubanks, unpubl. data). The selected outbred populations (OL and OH) had an unexpectedly high incidence of male sterility (apparently due to pleiotropic effects from selection on stigma-anther separation in this population), and mean pollen viability in these populations was significantly lower than the outbred controls (OC). Inbreeding also reduced male fertility in IL and IH relative to OC, but their fertility was similar to OL and OH. The variability in pollen viability across these subpopulations provided potentially useful material for exploring our questions about the role of pollen reward quality in determining pollinator visitation (Yeamans *et al*. 2014). Inbreeding commonly affects non-floral traits in *M. guttatus* as well. Inbred plants have reduced biomass (e.g. Dudash *et al*. 1997) and lower tolerance of herbivory (e.g. Carr and Eubanks 2002).

*Bombus impatiens* is native to the eastern USA, and is a highly generalized forager. We obtained commercial ‘class B’ *B. impatiens* Natupol^®^ hives (Koppert Biological Systems, The Netherlands) for use in our experiments. These hives arrived with a queen and about 45–60 workers. Hives were supplemented with a nectar substitute that the bees could access *ad libitum*. Exiting and entering the hive could be controlled so that the bees are free to forage only during the desired periods.

### Live plant arrays

#### Variation in pollen rewards 2011

In 2011 a greenhouse population of *M. guttatus* was created from the material described earlier. We germinated seeds from 21 to 38 families from all five subpopulations in group A (21 IL, 24 IH, 38 OC, 34 OL and 35 OH) and from 25 to 38 families from each subpopulation in group B (30 IL, 25 IH, 35 OC, 38 OL and 37 OH). Two seedlings from each family were transplanted into individual 3″ pot filled with Farfard III potting soil. Seedlings from group A were randomized on opposite sides of a greenhouse bench (blocks A1 and A2), 20 plants per tray. Similarly, a pair of seedlings from each family in group B was randomized on opposite sides of a second bench (blocks B1 and B2). Plants were bottom watered, and supplemental sodium vapour lights maintained an 18:6 L:D photoperiod. The total number of plants in the experiment was 627 (not all families produced at least two seedlings).

As the plants came into flower, anthers were collected from a single open flower on each plant and stored in lactophenol with analine blue. Viable pollen will stain dark blue with analine blue, and inviable pollen grains will not (Kearns and Inouye 1993). Two samples of 100 pollen grains were scored for viability under a compound microscope from each plant, and the mean proportion of stained pollen grains from the two samples was used as an estimate of pollen viability.

#### Analysis

All analyses were conducted with SAS 9.4. To determine whether pollen viability varied among subpopulations, we conducted a generalized linear mixed model using SAS proc glimmix. Because pollen viability was measured as a proportion (number viable grains/total grains), we assumed a binomial distribution and used a logit link function (Bolker *et al*. 2009). Subpopulation was included in the model as a fixed effect, and block (A1, A2, B1 and B2) and family nested within subpopulation were included as random effects.

#### Variation in pollinator visitation 2011

Once more than 80 % of the plants had come into flower, we introduced a *B. impatiens* hive into the greenhouse. The bees were allowed to forage on *M. guttatus* in the greenhouse for several days prior to the initial pollinator observations in order to acclimate them to the greenhouse. A few hours prior to an observation period, the total number of open flowers on each plant was recorded. At the onset of an observation period (beginning between 1300 and 1500 h), one observer was randomly stationed at each of the four blocks, and ∼12 bees were allowed to leave the hive. When a bee arrived at a block, the observer would follow it and record each plant visited and the number of flowers on the plant that the bee probed. A ‘probe’ was recorded if the bee stuck its entire head into the corolla tube. The observer would follow the bee until it left that side of the table. Once a bee left, the observer would wait until a new bee arrived, and observations would ensue until it left. The observation period ended after 30 min so that resources would not become depleted. A total of three observation periods (12–14 July) were conducted. By the last observation period, 88 % of the population was in flower.

#### Analysis

To test whether the number of bumble bee visits to plants varied among subpopulations, we calculated the total number of times a bee arrived at each plant during each observation period. We then conducted a repeated-measures analysis of covariance (ANCOVA) using SAS proc mixed with an unstructured variance–covariance matrix (selected based on lowest AIC). ‘Arrivals’ (square-root transformed) served as the dependent variable, and subpopulation, observation day and their interaction were treated as fixed effects. The number of flowers open on each plant during the observation period was included as a covariate. Block (containing spatial variation and variation among observers) and family nested within subpopulation were included as random effects. The individual plant served as the ‘subject’ in the analysis.

To determine whether the number of flowers probed by bees once they arrived at a plant differed among subpopulations, we calculated the mean number of probes per plant per observation period. We then conducted a repeated-measures ANCOVA with an unstructured variance–covariance matrix and a mean number of probes as the dependent variable. The model was identical to the model used to test for variations in arrivals (see above).

#### Variation in pollen rewards 2013

In 2013 we repeated the experiment, using only plants from group A. We germinated seed from 14 to 19 maternal families from each of the five subpopulations (17 IL, 14 IH and 19 each for OC, OL and OH), and five seedlings from each maternal family were transplanted into individual 3-inch pots. One seedling from each family was placed in a random order on each of five greenhouse benches (blocks A–E). The experiment included 424 individual plants. Soil and lighting were identical to the previous year.

As the plants began to flower, pollen was collected from an open flower at the second or third node and stored in lactophenol in aniline blue to quantify pollen viability as described for the 2011 live plant array experiment. Pollen viability for each plant was determined from a mean of 2–3 pollen samples per plant.

#### Analysis

To test whether pollen viability varied among subpopulations, we conducted a generalized linear mixed model using SAS proc glimmix, assuming a binomial distribution and using a logit link function. Subpopulation was included in the model as a fixed effect, and blocks (A–E) and family nested within subpopulation were included as random effects.

#### Variations in pollinator visitation 2013

Once over 80 % of the plants were in flower, we allowed *B. impatiens* from a commercial hive to forage in the greenhouse for 2 days *ad libitum* prior to observations to allow for acclimation. At the beginning of each observation day, we counted the number of open flowers on each plant and measured the corolla width of a randomly selected flower with a digital calliper. Observations began between 1300 and 1500 hours, with each observation trial lasted 1 h, with one observer randomly stationed at each of the five greenhouse tables (blocks). Each observer followed a single bee from when it arrived at the table until it left, as before, recording each plant it visited as well as the number of flowers probed on each plant during a visit. We conducted five observation periods beginning on 27 June and continuing on alternate days until 5 July. At the time of the final observation period, 92 % of the population was in flower.

#### Analysis

To test whether bumble bee visitation to plants varied among subpopulations, we used a repeated-measures ANCOVA in SAS proc mixed with a compound-symmetric variance–covariance matrix (selected based on lowest AIC) and ‘arrivals’ (square-root transformed) as the dependent variable. Subpopulation, observation day and their two-way interaction were included in the model as fixed effects. The number of open flowers each day and corolla width were included as covariates. Block (accounting for spatial and observer variation) and family within subpopulation were included as random effects. Individual plants served as the ‘subject’ in the analysis.

To test whether the number of flowers probed after arrival to a plant varied among subpopulations, we calculated the mean number of flowers probed for each plant for each observation day. This served as the dependent variable in a repeated-measures ANCOVA using SAS proc mixed. The model was the same as described above for the dependent variable ‘arrivals.’

### Artificial flower arrays

**Figure 1.**
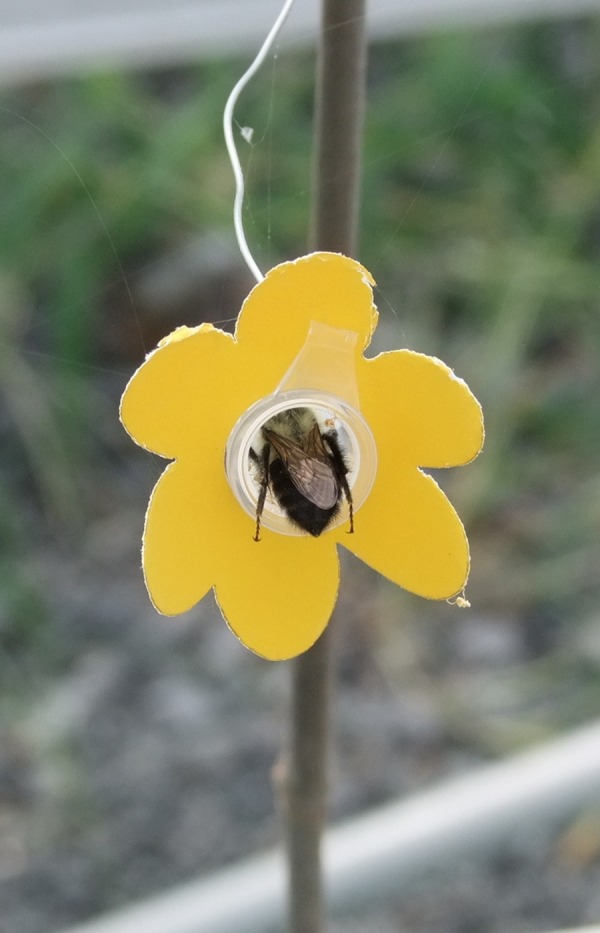
A *Bombus impatiens* visits an artificial flower provisioned with fresh *M. guttatus* anthers.

To determine whether bumble bees could discriminate between fertile and sterile *M. guttatus* anthers, we constructed arrays of artificial flowers. The artificial flowers (Fig. 1) were made from yellow construction paper (Y-HUE from Color Aid Corporation, Hudson Falls, NY, USA) cut into six-petal shapes with a ‘Cuttlebug Scribble Flower™’ die (Provo Craft and Novelty, Inc., South Jordan, UT, USA) and a Sizzix^®^ press (Lake Forest, CA, USA). The cap was removed from a 1.5 mL clear microcentrifuge tube, and it was inserted through a hole cut in the centre of the petals to act as a corolla tube. Four of these artificial flowers were attached to a thin bamboo stake by wire pedicles to make an artificial plant. The bamboo ‘stems’ were anchored in a 3-inch pot filled with gravel such that the whorl of four artificial flowers stood at a height of ∼30 cm. A total of 24 artificial plants were constructed and arranged on greenhouse benches in a 6 × 4 matrix.

At the beginning of each trial we collected fresh anthers from newly opened *M. guttatus* flowers. We randomly assigned the anthers to artificial plants (four anthers per artificial flower, four flowers per plant), such that six plants were provisioned with anthers containing high viability outcross pollen, six with low viability outcross pollen, six with high viability inbred pollen and six with low viability inbred pollen. Pollen was collected from the same plants used in the 2011 live plant arrays. High viability was defined as >80 % viable, and low viability ranged from 0 to 50 % viable. A single observer conducted observations during eight different periods beginning between 0900 and 1000 hours from 19 to 31 July 2011, using fresh pollen and a new randomization each period. Observation periods lasted from 1.5 to 2 h, depending on bee activity.

#### Analysis

To test the null hypotheses that bee arrival at artificial plants was independent of reward quality (low versus high viability pollen) and whether the pollen came from inbred or outbred plants, we conducted a log-linear model with SAS proc catmod. The model included both main effects and their interaction. The data were pooled across the 4 observation days.

To test whether the mean number of probes differed between artificial plants provisioned with low or high viability pollen or between artificial plants provisioned from inbred or outbred plants, we conducted a factorial ANOVA with SAS proc mixed. Pollen viability (low or high), inbreeding level (inbred or outbred) and their interaction were treated as fixed effects and data were pooled across the 4 observation days.

### Olfactometer tests

#### Fertile versus sterile anthers

To test whether bumble bees had a preference for fertile anthers based on olfactory cues alone, we conducted a pairwise choice experiment with free-flying *B. impatiens* in the greenhouse. In July 2009 we constructed two artificial flowers that served as part of an olfactometer device. These flowers were constructed as described in the Artificial Flower Array experiment except that they were the only flowers attached to their bamboo stems and the bottoms of the microcentrifuge tubes were clipped to provide an opening for airflow from an odour source. The bottoms of the microcentrifuge tubes were connected by Teflon^®^ tubing to one of two 5-L ARS^®^ volatile chambers (ARS, Gainesville, FL, USA). One of the chambers contained 40 fertile anthers from outbred *M. guttatus* suspended on a screen to allow air to pass through them. The other contained 40 sterile anthers from outbred *M. guttatus* on a screen. A tank of compressed air was connected to an ARS 2-channel Air Delivery System (ARS, Gainesville, FL, USA) that split the airflow into two equal pressure streams through the volatile chambers and out to the artificial flowers. The difference in volatile cues emitted by the olfactometer flowers therefore would be determined solely by the volatile chemical difference between the sterile and fertile anthers in the chambers.

In addition to the two olfactometer flowers, we created a small population of 12 additional artificial plants, each with four artificial flowers. The artificial flowers in half of these plants were each provisioned with four fertile anthers freshly collected from *M. guttatus* and the other half with four sterile anthers. These artificial plants simply served to attract bees to the greenhouse bench housing the olfactometer flowers. The olfactometer flowers were provisioned with four small, anther-sized bits of yellow foam to provide a visual cue, but no actual reward. We monitored bee visitation to the olfactometer flowers for 1 h trials on 3 consecutive days (23–25 July). The *B. impatiens* used in this experiment had experience foraging on live *M. guttatus*, but no live plants were available to them during the trial periods.

#### Analysis

To test the null hypothesis that bees randomly visited the fertile and sterile olfactometer flowers, we conducted a χ^2^ goodness-of-fit test with SAS proc freq. The data from the three observation periods were pooled.

#### Inbred versus outbred plants

The ability of bumble bees to discriminate between inbred and outbred plants based on their volatile signals alone was evaluated using a Y-tube experiment on 25 July 2013. The *B. impatiens* used in this study had never foraged on *M. guttatus* before. A pair of inbred and outbred *M. guttatus* (matched for equal numbers of open flowers but otherwise randomly selected) from the 2013 live plant array experiment was placed into paired, glass 5-L ARS^®^ volatile chambers and connected by Teflon^®^ tubing to either side of a Y-tube. The flowers were isolated in the airflow by an aluminium foil collar around the plant in order to minimize the contribution of foliar volatiles. The inbred and outbred plants were randomized to the left or right forks of the Y prior to each individual trial. Airflow through the chamber and into the Y-tube was generated with a Syntech Stimulus Controller CS-55 set at its lowest setting. A single bee was introduced into the proximal end of the 30-cm Y-tube and allowed to crawl towards the bifurcation. During the trial, most of the Y-tube was covered with a black felt sheet to minimize the effect of lighting and other extraneous stimuli in the lab. A bee was scored as choosing an arm if it crossed a line 5 cm from the bifurcation. Individual bees were used only once in the experiment. A total of five pairs of inbred and outbred plants were used in the experiment, introducing a new pair after eight to nine trials. Four bees failed to make a choice within 10 min and were omitted from the analysis.

#### Analysis

To test the null hypothesis that bees chose inbred and outbred plants at random in the olfactometer experiment, we conducted a log-linear model using SAS proc catmod. The model included the level of inbreeding in the plants (inbred or outbred), the pair of plants used in the experiment (pairs 1–5) and their two-way interaction.

## Results

### Live plant arrays

**Figure 2.**
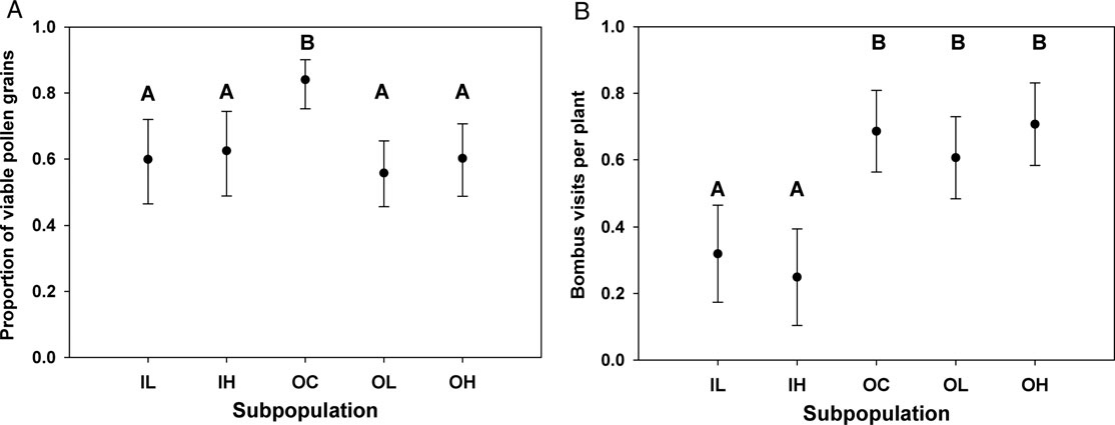
(A) Mean pollen viability in the five *M. guttatus* subpopulations in the 2011 live plant experiment. Estimates represent least-squares means derived from a generalized mixed model, back transformed from the odds ratios. Error bars indicate 95 % confidence intervals. Means with different letters are significantly different based on a Tukey–Kramer test. (B) Mean *B. impatiens* visits per plant per day in the five subpopulations in the 2011 live plant experiment. Estimates represent least-squares means derived from a mixed model ANCOVA. Error bars indicate 95 % confidence intervals. Means with different letters are significantly different based on a Tukey–Kramer test.

In the 2011 live plant array experiment, pollen viability differed significantly across subpopulations (Table 1a). Pollen viability in the unselected controls (OC) was 26–34 % higher than any of the other subpopulations (Fig. 2A), indicating superior reward quality. The selected inbred (IL and IH) and outbred (OL and OH) subpopulations did not differ significantly in pollen viability, indicating that reward quality was comparable.

**Table 1.** Analysis of pollen viability and pollinator responses in the 2011 live plant arrays. Included are the tests of the null hypothesis that there is no variation among our *M. guttatus* subpopulations (IL, IH, C, OL and OH) in (a) pollen viability, (b) bumble bee arrivals to plants and (c) the number of flowers visited per arrival from the 2011 live plant array. The GLMM used a binomial distribution and logit link to analyze the proportion of viable pollen grains per plant. The repeated-measures mixed model ANCOVAs used an unstructured variance–covariance structure. Fixed effects were tested with *F*-ratios. Random effects were tested with 1 df log-likelihood ratio tests (*G*).

Effect	df	ddf	Fixed or random	*F*	Variance component	*G*	*P*
(a) 2011 Pollen viability generalized linear mixed model							
Subpopulation	4	400	Fixed	5.20	–	–	0.0004
Block	3	–	Random	–	0.027	0.79	0.1871
Family (subpop)	400	–	Random	–	0.000	0.00	1.0000
(b) 2011 Bumble bee visitation general linear mixed model RMANCOVA							
Subpopulation	4	297	Fixed	12.78	–	–	>0.0001
Day	2	1356	Fixed	28.46	–	–	>0.0001
Flowers	1	1356	Fixed	2.16	–	–	0.1417
Subpop × day	8	1356	Fixed	0.85	–	–	0.5596
Block	4	–	Random	–	0.001	1.90	0.0840
Family (subpop)	297	–	Random	–	0.000	0.00	1.0000
(c) 2011 Floral visits general linear mixed model RMANCOVA							
Subpopulation	4	246	Fixed	0.56	–	–	0.6949
Day	2	357	Fixed	0.47	–	–	0.6249
Flowers	1	357	Fixed	8.35	–	–	0.0041
Block	4	–	Random	–	0.004	0.90	0.1714
Family (subpop)	246	–	Random	–	0.015	0.40	0.2635

We observed a total of 1726 pollinator arrivals over the course of the 3 observation days. Arrivals varied significantly among subpopulations (Table 1b), but the pattern (Fig. 2B) did not match the pollen viability pattern. Bumble bees visited plants from outbred subpopulations approximately twice as often (1.9–2.5×) as inbred subpopulations despite the fact that mean pollen viability in the OL and OH subpopulations was equivalent to both of the inbred subpopulations. The visitation to the OC subpopulation did not differ significantly from the other outbred subpopulations, despite its much higher pollen viability. Once bees arrived at a plant, they probed about 1.4 flowers, on average (s.e. = 0.12), and this did not differ significantly across subpopulations (Table 1c).

**Figure 3.**
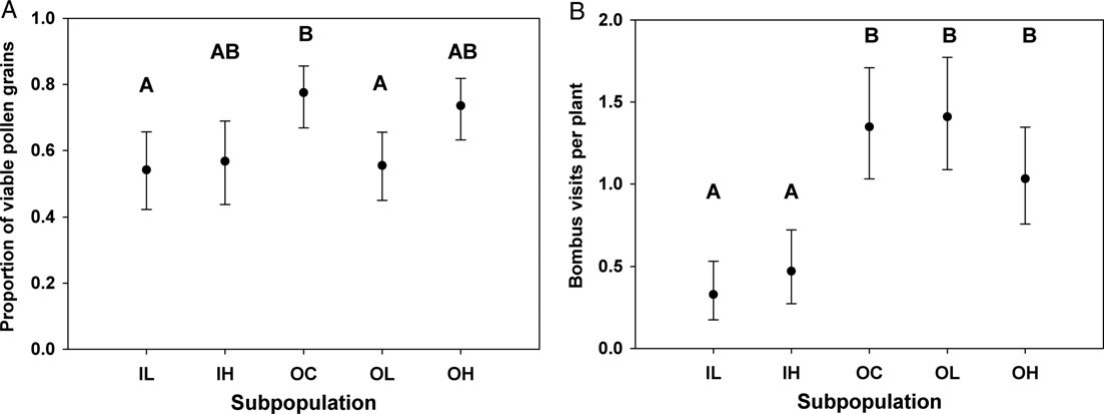
(A) Mean pollen viability in the five *M. guttatus* subpopulations in the 2013 live plant experiment. Estimates represent least-squares means derived from a generalized mixed model, back transformed from the odds ratios. Error bars indicate 95 % confidence intervals. Means with different letters are significantly different based on a Tukey–Kramer test. (B) Mean *B. impatiens* visits per plant per day in the five subpopulations in the 2013 live plant experiment. Estimates represent least-squares means derived from a mixed model ANCOVA. Error bars indicate 95 % confidence intervals. Means with different letters are significantly different based on a Tukey–Kramer test.

We repeated the live plant array experiment in 2013, restricting our greenhouse population to subpopulations from group A. Pollen viability varied significantly among the subpopulations (Table 2a), but the pattern (Fig. 3A) was more complex in this experiment than that in 2011. Pollen viability in subpopulation OC was significantly higher than IL and OL (30 and 29 %, respectively), but IH and OH did not differ significantly from OC. Subpopulations IL, IH, OL and OH did not differ significantly from one another.

**Table 2.** Analysis of pollen viability and pollinator responses in the 2013 live plant arrays. Included are the tests of the null hypothesis that there is no variation among our *M. guttatus* subpopulations (IL, IH, C, OL and OH) in (a) pollen viability, (b) bumble bee arrivals to plants and (c) the number of flowers visited per arrival from the 2013 live plant array. The GLMM used a binomial distribution and logit link to analyse the proportion of viable pollen grains per plant. The repeated-measures mixed model ANCOVAs used a compound-symmetric variance–covariance structure. Fixed effects were tested with *F*-ratios. Random effects were tested with 1 df log-likelihood ratio tests (*G*).

Effect	df	ddf	Fixed or random	*F*	Variance component	*G*	*P*
(a) 2013 Pollen viability generalized linear mixed model							
Subpopulation	4	82	Fixed	4.23	–	–	0.0037
Block	4	–	Random	–	0.010	0.4	0.2635
Family (block × subpop)	82	–	Random	–	0.000	0.0	1.0000
(b) 2013 Bumble bee visitation general linear mixed model RMANCOVA							
Subpopulation	4	82	Fixed	17.60	–	–	>0.0001
Day	2	1632	Fixed	28.80	–	–	>0.0001
Flowers	1	1632	Fixed	79.04	–	–	>0.0001
Corolla width	1	1632	Fixed	1.72	–	–	0.1902
Subpop × day	16	1632	Fixed	1.07	–	–	0.3827
Block	4	–	Random	–	0.008	6.2	0.0064
Family (subpop)	297	–	Random	–	0.011	4.3	0.0191
(c) 2013 Floral visits general linear mixed model RMANCOVA							
Subpopulation	4	81	Fixed	2.52	–	–	0.0471
Day	2	967	Fixed	3.57	–	–	0.0067
Flowers	1	967	Fixed	18.18	–	–	<0.0001
Corolla width	1	967	Fixed	0.01		–	0.9147
Block	4	–	Random	–	0.008	0.7	0.2014
Family (subpop)	81	–	Random	–	0.038	3.5	0.0307

In 2013 we observed 1098 pollinator arrivals across the five observation periods. Again arrivals varied significantly among our subpopulations (Table 2b), but again the pattern did not match the pattern of variation in reward quality. Each outbred subpopulation received at least twice as many *B. impatiens* visits as either of the two inbred populations (Fig. 3B) despite the fact that pollen viability in OL was essentially equivalent to the pollen viability in each of the inbred subpopulations. Visitation did not differ significantly among outbred populations despite the fact that OL had significantly lower pollen viability than OC.

In 2013, subpopulation had a relatively small effect on the number of flowers bumble bees probed once they arrived on a plant (Table 2c). Bees visited significantly fewer flowers when arriving at IL plants (1.40 ± 0.13) compared to the OC plants (1.86 ± 0.09) based on a Tukey–Kramer multiple comparison test, but no other pairwise comparisons showed significant differences.

In both years, the patterns of arrivals were consistent throughout the days of observation (i.e. the subpopulation × day interactions were not significant, Tables 1b and 2b), indicating no change in preference during the course of the experiments. Bees also did not appear to respond to differences in trichome density within the inbred or outbred populations in either year because there was never a difference in visitation rates between high and low trichome lines within either inbred or outbred plants (Figs 2b and 3b).

### Artificial plant arrays

**Figure 4.**
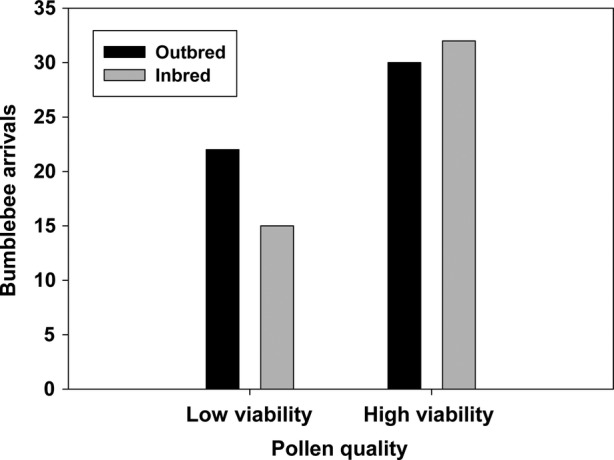
*Bombus impatiens* visitation to artificial plants with flowers provisioned with either low viability pollen from outbred *M. guttatus* plants, low viability pollen from inbred plants, high viability pollen from outbred plants or high viability pollen from inbred plants.

We observed a total of 99 bumble bee arrivals to artificial plants and 193 probes into artificial flowers. Bumble bees were 68 % more likely to visit artificial plants provisioned with high viability pollen relative to low viability pollen (*χ*^2^ = 6.45, *P* = 0.0111; Fig. 4). Bees were no more likely to visit artificial plants provisioned with pollen from outbred plants than those provisioned from inbred plants (*χ*^2^ = 0.57, *P* = 0.4487), and the attraction to high viability pollen was independent of whether the pollen came from inbred or outbred plants (*χ*^2^ = 1.13, *P* = 0.2870).

The mean number of artificial flowers probed once a bee arrived at a plant was slightly higher for plants provisioned with high viability pollen (2.08 ± 0.13) relative to plants provisioned with low viability pollen (1.76 ± 0.17), but the difference was not significant (*F*_1,95_ = 2.30, *P* = 0.1326). The mean number of probes was not different between artificial flowers provisioned with pollen from inbred or outbred plants (*F*_1,95_ = 0.17, *P* = 0.6791), and the effect of pollen viability was independent of the level of inbreeding (*F*_1,95_ = 1.45, *P* = 0.2323).

### Olfactometer tests

In our test for a preference for olfactory cues from fertile or sterile anthers, only 22 free-flying *B. impatiens* visited the ‘olfactometer’ flowers over the course of the three observation periods. Despite the low sample size, 16 (73 %) of these bees visited fertile anthers, demonstrating a significant bias in visitation (*χ*^2^ = 4.55, 1 df, *P* = 0.0330).

A total of 41 *B. impatiens* were used in our pairwise olfactometer choice tests for inbred and outbred plants. Four of those bees made no choice in the Y-tube. Of the 37 bees that made a choice, 29 (78 %) chose the outbred plant (*χ*^2^ = 10.28, 1 df, *P* = 0.0013), and the preference for outbred plants was consistent across the five different pairs of inbred and outbred plants used in the experiment (inbreeding × pair interaction: χ^2^ = 2.09, 1 df, *P* = 0.7187).

## Discussion

The central importance of pollen as a protein source for bees should generate strong selective pressures for the ability to efficiently locate the best sources of this resource. Inviable pollen grains appear to contain little or no protein in *M. guttatus* (Yeamans *et al*. 2014), indicating that reduced viability will reduce the protein award available to a visiting pollinator. Our data demonstrated that *B. impatiens* is capable of discriminating between fertile and sterile anthers when presented with cues from anthers in isolation from other cues provided by the entire flower. This ability could allow bees to spend most of their time and effort foraging on the most rewarding flowers. However, we found that bees seem less capable of making distinctions based on pollen reward quality when foraging among live, intact plants.

Bumble bees made no distinction between outbred subpopulations that differed dramatically in their mean pollen viability. The lack of discrimination between better and less rewarding plants is consistent with an earlier report that *B. impatiens* did not significantly discriminate between male fertile and male sterile *M. guttatus* (Wise *et al*. 2011). Carr *et al*. (2014) found that bumble bee visitation increased with pollen viability in only one of the two populations, but even in this population, pollen viability explained only ∼8 % of the variation in visitation. In contrast, Robertson *et al*. (1999) did find that bumble bees preferred higher viability pollen when given pairwise choices between *M. guttatus* plants or choices between large patches of plants. They were unable, however, able to demonstrate a preference for plants producing high viability pollen when the bees foraged in a mixed population of good and poor pollen producers, an experimental design similar to that used here, in Wise *et al*. (2011) and in Carr *et al*. (2014).

We demonstrated that *B. impatiens* strongly discriminated against inbred *M. guttatus*, a finding consistent with earlier studies (Ivey and Carr 2005; Carr *et al*. 2014). This discrimination was evident even when outbred plants had, on average, pollen viability as low as that in inbred plants. Inbreeding reduces traits in *M. guttatus* that are known to be attractive to pollinators, including corolla size (Ivey and Carr 2005), flower number (Dudash *et al*. 1997), as well as pollen rewards, but we found here and elsewhere (Ivey and Carr 2005; Carr *et al*. 2014) that the preference for outbred plants persisted even when controlling for corolla size and floral display size in the analyses. The bees also did not show any preference for anthers collected from inbred or outbred plants in our artificial flower experiments, suggesting that the basis of their discrimination lies elsewhere.

We also demonstrated that bumble bees manifested a preference for outbred plants even when they were provided with only olfactory cues. It is interesting to note that this preference existed even though these bees had never foraged on *Mimulus* before. Olfactory cues have been shown to be important in bumble bee preference for *Mimulus lewisii* over the closely related, hummingbird-pollinated *M. cardinalis* (Byers *et al*. 2014*a*, *b*), and Parachnowitsch *et al*. (2013) demonstrated that pollinator-mediated natural selection on floral scent in *Penstemon digitalis* was more intense than selection on floral morphology or colour. Our data certainly suggest that volatile signals could play an important role in the pollination biology of *M. guttatus* and bumble bee preference for outbred plants. Inbreeding in *Cucurbita pepo* alters floral volatile emissions (Ferrari *et al*. 2006), and the emission of foliar volatiles is altered by inbreeding in *Solanum carolinense* with consequences for insect behaviour (Kariyat *et al*. 2012, 2013, 2014). We could not find any published studies of inbreeding effects on volatile production in *Mimulus*.

*Bombus impatiens* showed a preference for fertile (rewarding) anthers based on olfactory cues alone. Volatile compounds that are unique to pollen and anthers (Dobson and Bergström 2000; Ashman *et al*. 2005) or otherwise are correlated with rewards could serve as ‘honest’ signals that can indicate to pollinators the relative value of visiting a particular flower (Raguso 2008; Ashman 2009; Wright and Schiestl 2009). If so, our inability to demonstrate a bumble bee preference for *M. guttatus* from the most rewarding subpopulations seems paradoxical. Although honest signalling may benefit pollinators by enabling more efficient foraging, honesty may not always be the best policy for plants if plant populations comprise morphs that differ in their pollen rewards (e.g. gynodioecious or dioecious species) or if they benefit from additional visits (e.g. Karron *et al*. 2006) even after rewards are depleted. In these cases, selection may favour plants that somehow obfuscate signals that are highly correlated with reward status. While deceptive pollination syndromes based on mimicry or the exploitation of sensory biases are well documented in rewardless orchids, for example (Alcock 2005; Schiestl 2005; Schaefer and Ruxton 2009), more subtle levels of deception have not been explored. The differences that we observed in pollinator response to live plants relative to isolated anthers suggest that the information transmitted from flowers to pollinators, at the very least, is not straight forward.

## Conclusions

The complexity of plant–pollinator interactions is becoming more evident as our exploration goes deeper (e.g. Adler and Bronstein 2004; Kessler and Baldwin 2007; Praz *et al*. 2008; Irwin *et al*. 2010). The genus *Mimulus* seems to offer the ecological diversity combined with an experimental accessibility necessary to be an important contributor to furthering our understanding of these interactions from the genetic to the evolutionary and from the biochemical to the behavioural. Our relatively simple experiments thus far have demonstrated that bumble bee preference in *M. guttatus* can be independent of reward quality and have suggested that its flowers use highly interactive and perhaps even deceptive cues in attracting its pollinators.

## Sources of Funding

This research was supported by grants from the US National Science Foundation (DEB-0614395, DEB-0828892, DBI-1034846 and DBI-1156796).

## Contributions by the Authors

D.E.C. worked with all co-authors to design experiments and in data collection. He was responsible for data analysis. A.I.H. conducted olfactometer tests and assisted with data collection on live plant arrays in 2013. K.A.L. performed pollen viability assays and conducted live plant arrays in 2011. D.E.L. performed pollen viability assay and conducted live and artificial plant arrays in 2011. R.I.L. conducted the live plant array experiment, performed viability assays and worked with the olfactometer experiments in 2013. D.E.C. wrote the first draft of the manuscript, and all co-authors contributed editorial comments.

## Conflict of Interest Statement

None declared.
